# Impact of a Six-Week Prehabilitation With Blood-Flow Restriction Training on Pre- and Postoperative Skeletal Muscle Mass and Strength in Patients Receiving Primary Total Knee Arthroplasty

**DOI:** 10.3389/fphys.2022.881484

**Published:** 2022-06-14

**Authors:** Alexander Franz, Sanghyeon Ji, Bernd Bittersohl, Christoph Zilkens, Michael Behringer

**Affiliations:** ^1^ Department of Orthopedics and Trauma Surgery, University Hospital Bonn, Bonn, Germany; ^2^ Department of Adult Reconstruction, ATOS Orthoparc Clinic Cologne, Cologne, Germany; ^3^ Department of Exercise Physiology, Institute of Exercise Training and Sport Informatics, German Sport University Cologne, Cologne, Germany; ^4^ Department of Orthopedics and Trauma Surgery, University Hospital Duesseldorf, Duesseldorf, Germany; ^5^ Department of Sports Medicine and Exercise Physiology, Goethe University Frankfurt, Frankfurt, Germany

**Keywords:** venous occlusion, kaatsu training, muscle atrophy, rehabilitation, exercise therapy

## Abstract

**Introduction:** Total Knee Arthroplasty (TKA) is one of the most successful interventions in gonarthrosis, however the operation is leading to muscle atrophy and long-term muscular deficits. To enhance rehabilitation after TKA, exercise programs try to improve muscle function preoperatively, called prehabilitation. Blood-Flow-Restriction Exercises (BFRE) is a training method which is characterized by using tourniquets to reduce arterial and occlude venous blood flow simultaneously during the exercise to increase metabolic stress. The present study aimed to evaluate the effects of a 6-week prehabilitation with BFR on pre- and postoperative muscle mass, strength, and quality of life (QoL).

**Methods:** 30 patients with end-stage gonarthrosis participated in this study. Patients were randomized into one of three groups: 1) Control-Group (CON): Standard clinical approach without prehabilitation. 2) Active-Control-Group (AC): Participation in a prehabilitation with sham-BFR. 3) BFR-Group (BFR): Participation in a prehabilitation with BFR. The prehabilitation protocol consist of a cycling-ergometer-based training performed twice per week over 6 weeks. During exercise, BFR was applied periodically three times per leg with a pressure of 40% of the individual-limb-occlusion-pressure. Measurement time points were six- (baseline), 3-weeks and 5-days before the surgery (Pre-OP), as well as three- and 6-months postoperatively. Outcome measures were muscular strength of the thigh muscles, thigh circumference as well as QoL and functional activity, examined by 6-min walking- and chair rising test.

**Results:** Both training groups indicated significantly improved leg muscle strength following the prehabilitation period with a superior effect for the BFR-group (BFR: ∼170% vs. AC: ∼91%, *p* < 0.05). No significant changes in leg strength occurred in the CON (∼3%, *p* = 0.100). Further, patients in BFR-group indicated significantly improved skeletal muscle mass assessed by femoral circumference following prehabilitation period (∼7%, *p* < 0.05), while no significant changes occurred in the CON (−1.14%, *p* = 0.131) and AC-group (∼3%, *p* = 0.078). At 3-months Post-OP, the CON and BFR-group revealed a significant decrease in femoral circumference compared to the Pre-OP (CON: ∼3%, BFR: ∼4%; *p* < 0.05), but BFR-group remained above the baseline level (∼3%, *p* < 0.05). No significant change in femoral circumference was found for AC-group (∼2%, *p* = 0.078). In addition, the prehabilitation with BFR provided notably improved Knee Injury and Osteoarthritis Outcome Scores (KOOS) especially in pain perception with significant higher effect compared to other groups (CON: −2%, AC: 13%, BFR: 41%; *p* < 0.05). In long-term rehabilitation after 6-months, all groups showed significantly improved KOOS scores in all dimensions (CON: ∼110%, AC: ∼132%, BFR: ∼225%; *p* < 0.01), and functional examinations (CON: ∼26%, AC: ∼16%, BFR: ∼53%; *p* < 0.01).

**Conclusion:** The present findings show that BFR-prehabilitation induce significant improvements in muscle function and QoL before TKA surgery. In addition, the supporting effect of prehabilitation on postoperative regeneration and QoL should be highlighted, illustrating prolonged beneficial effects of BFR on muscular and functional performance in a “better in, better out”-manner.

## Introduction

Total Knee Arthroplasty (TKA) is one of the most popular and successful interventions in gonarthrosis leading to significant improvements in subjective pain and quality of life (QoL) (Vos et al., 2012). However, knee osteoarthritis (OA) as well as surgical therapy have adverse effects on skeletal muscle mass and strength (Franz et al., 2019). Predominantly due to pain-related reductions in mobility and exercise, patients receiving TKA are characterized by reduced muscular function preoperatively (Dreyer et al., 2013). Although postoperative rehabilitation shows a significant impact on patient mobility and QoL, recent meta-analyses show that TKA patients are affected by persistent muscle weakness and atrophy for several years (LaStayo et al., 2009; Thomas and Stevens-Lapsley, 2012).

Since physical patient characteristics like muscle mass, strength and functionality can be seen as positive predicate outcomes parameters for a successful rehabilitation (Mizner RL. et al., 2005; Devasenapathy et al., 2019), several studies try to support rehabilitation after TKA by improving muscle function already preoperatively through exercise, called prehabilitation. Unfortunately, common training techniques cannot provide an adequate stimulus for muscular adaptations without provoking increased pain (Juhl et al., 2014). Consequently, the current impact of available prehabilitation concepts is rated as only slight to moderate (Wang et al., 2016; Moyer et al., 2017).

Blood-Flow-Restriction Exercises (BFRE) are a new training method that is characterized by the use of specialized tourniquets to restrict venous and reduce arterial blood flow during the exercise in the working limb to increase metabolic stress. Since BFRE can gain significant effects on muscle mass and strength by using only low mechanical loads (Ferraz et al., 2018; Franz et al., 2020) its application in patients with degenerative joint diseases could be able to improve the applicability and effectiveness of prehabilitation concepts (Franz et al., 2018; Žargi et al., 2018; Kacin et al., 2021).

Therefore, the present study aimed to assess the impact of a 6-week prehabilitation protocol with BFRE on pre- and postoperative muscle mass, strength, functionality and subjective pain perception in patients receiving an elective primary TKA.

## Methods

### Subjects

30 patients suffering from end-stage gonarthrosis (male = 18, female = 12, age = 63.5 ± 8.1 y, height = 176.4 ± 7.2 cm, weight = 86.9 ± 16.1 kg) participated in this study. Patients were randomly assigned into one of three groups: 1) Control-Group (CON): This group followed the standard clinical treatment without a specialized prehabilitation protocol. 2) Active-Control-Group (AC): The second group followed the standard clinical treatment and participated in a 6-week prehabilitation protocol with a sham-BFR application. 3) BFR-Group (BFR): The third group followed the standard clinical treatment and participated in a 6-week prehabilitation protocol with additional BFR application. The standard clinical treatment consists of the surgery and 7 days of hospitalization with daily physical therapy, which was followed by 3 weeks of inpatient rehabilitation.

### Study Design

The study design consists of a prospective, single blinded, parallel study design to determine the influence of prehabilitation on pre- and postoperative skeletal muscle mass and strength. While the CON underwent routine clinical practice without prehabilitation, the other groups completed an identical 6-week prehabilitation protocol, one with additional BFRE (BFR-Group) and one with a sham-BFR application (AC-Group) before TKA, to reduce a potential bias in expectations of the intervention effect between groups. Preliminary visits were conducted before the start of the study to familiarize the patients with the cycling ergometer, testing protocols and tourniquet pressures.

Measurement time points were 6 weeks- (baseline), 3 weeks- (3w-Prehab) and 5 days before surgery (Pre-OP), as well as 3- (3m-Post-OP) and 6-months (6m-Post-OP) postoperatively.

#### Prehabilitation

The prehabilitation protocol consist of a cycling ergometer-based training protocol performed twice per week for about 50 min with an individualized intensity. Ergometer intensity was determined based on a calculated exercise heart rate (EHR) (Mangione et al., 1999).

For determination of EHR, maximal heart rate [HR (max) = 220—Age) and heart rate reserve (HRR = HR (max)—HR (rest)] of each participant was calculated. Subsequently, the EHR was determined by the following calculation model:

EHR = HR(rest) + (HRR x 0.5)

The load in watts matching the calculated EHR was determined during an incremental step test on the cycling ergometer. The test person starts at an intensity of 20 W (W) on the cycling ergometer, which was increased by 20 W every 3 minutes. Vital signs such as blood pressure (RR) and HR are determined at the beginning and end of each stage. As soon as the test person has reached the calculated EHR, the test was finished.

**FIGURE 1:**
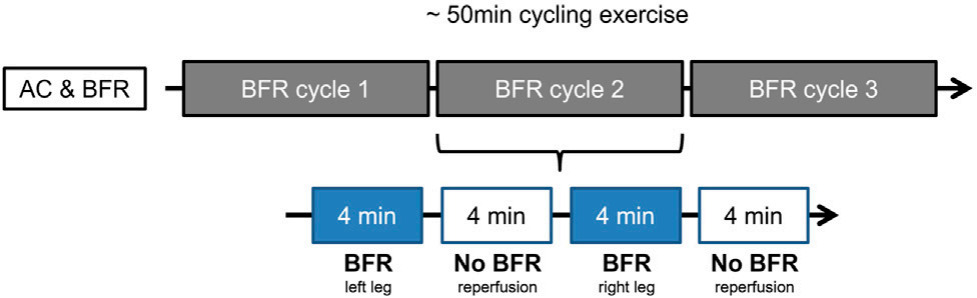
Example of prehabilitation week four for AC- and BFR-group. The 50-min ergometer exercise is divided into three cycles. Each cycle consists of the application of the BFR stimulus to both legs in alternation, followed by a rest. As shown here, the left leg is loaded with BFR first (for 4 minutes in week four), followed by a rest of 4 min. This is followed by BFR application to the right leg with a subsequent break. This cycle is repeated a total of three times.

The additional BFRE protocol was applied during the cycling exercise on both lower limbs periodically three times per leg for a duration of 1 min (first week) to 6 min (sixth week). While the AC group performed a sham-BFR exercise with a fixed value of 20 mmHg, the BFR-group was loaded with 40% of the individual limb occlusion pressure (LOP; right = 88.27 ± 8.46 mmHg, left = 87.32 ± 7.39 mmHg) (Figure 1).

To determine the LOP, the inflatable tourniquets of 11.5 cm width were placed proximal at the exercising legs before the training session (PBFR, Delfi medical Inc., Vancouver, Canada). After a 10-min rest period, LOP was determined sonographically in a lying position by displaying the femoral artery with an ultrasound device and using a Doppler to assess the blood flow within the vessel. Subsequently, the cuff was infiated until no further blood flow was detectable. This pressure was defined as the individual LOP.

#### Outcomes Measures

The examination battery consisted of general vital and anthropometric data, muscular function parameters, functional examinations, and questionnaire-based data collection.

### Vital- and Anthropometric Data

At each time point, vital parameters like blood pressure (RR) and heart rate (HR) were recorded. Anthropometric data consist of recording body weight (BW), body height (BH) as well as the circumference of both thighs and calves for estimation of skeletal muscle mass of the lower extremities. To visualize the femoral circumference an anatomic reference line was drawn between the spina iliaca anterior superior and the margo superior patella. At 50% (FC-50) length of this reference line, the femoral circumference of both legs was determined. The calf circumference was determined after multiple measurements at the largest diameter. The measurement was performed three times at all points and the mean value was determined. Furthermore, the circumference of both knees was recorded to illustrate swelling pre- and postoperative. Measurement points were above the margo superior patellae, in the middle of the patella as well as above the apex patellae.

### Muscular Strength

Muscular function in this study was analyzed by a unilateral six-repetition maximum test (6RM) of the leg-extension and leg-curl machine (Paoli et al., 2013). Following a warmup, a maximum of five trials separated by 5 min of recovery was allowed to obtain a true 6RM. With an accuracy of 1.00 kg, the highest load that the subject was able to lift six times to a knee extension of approximately zero degrees was accepted as 6RM.

### Assessment of Function

The functionality of the participating patients was determined by active knee joint mobility (range of motion, ROM) as well as the chair-rising- (CRT) and 6-min walking test (6MWT). The ROM of the knee joint was determined in the supine position. A goniometer was used to measure the active extension and flexion of the knee joint (Bade et al., 2014). In CRT, the subject sits on an ordinary chair without armrests. With arms crossed on the chest, the test person performs as many stand-up and sit-down movements as he or she can manage within 30 s (Gill and McBurney, 2008). A complete repetition was then scored after the test person was in full extension while standing, as well as with a leaning back in a sitting position. The 6MWT is used to estimate and monitor cardiovascular and pulmonary performance below the anaerobic threshold. Participating patients walk for 6 minutes along a 20 m walkway. The goal for the patient is to complete as much distance as possible in the given time window. Individual pace changes and pauses are allowed. After 6 minutes, the time is stopped, and the walking distance is written down in meters.

### Patient Self-Assessment Tools

To assess the subjectively experienced functional status, pain perception and quality of life of the patients, the Knee Injury and Osteoarthritis Outcome Score (KOOS) was used. KOOS contents of 42 questions in five different dimensions: Symptoms (seven questions), pain (nine questions), activities of daily living (17 questions), functionality in sports and recovery (five questions) and quality of life-related to the affected knee (four questions).

### Statistics

Statistical analyses were performed using SPSS (SPSS, v.27, Chicago, IL, United States). Normal distribution and homogeneity of variance were verified using Shapiro-Wilk and Levene’s Test, respectively. Potential group differences in baseline and surgical data (i.e., age, height, body weight, duration of surgery, etc.) were assessed using one-way ANOVAs. To compare the changes in measures over time among groups, two-way repeated-measures ANOVAs (rANOVAs; time x group) were performed. In case of significant time × group interactions, separate one-way repeated measures ANOVAs were used to analyze the simple main effects for time within each group. The mean differences between groups (i.e., simple main effects for group) within each time point were assessed using separate one-way ANOVAs. If the main effects for time or group were detectable, *post hoc*-tests with Bonferroni correction were performed to check which factor levels differ significantly from one another. For the interaction and main effects, the partial eta squared η_p_^2^ was calculated as effect strength measure and interpreted as follows (Cohen, 1988): a η_p_^2^ ≥ 0.01: small effect, ≥ 0.06: medium effect, and ≥0.14: large effect. For all results, an alpha level of 0.05 was interpreted as statistically significant. To reduce investigator bias, data analysis was blinded to the evaluating researchers.

## Results

Table 1 presents the baseline and surgical data for each group. At baseline, there were no significant differences regarding their demographic data. In addition, the mean duration of surgery and blood loss during surgery did not differ among all groups.

**TABLE 1:** Patient characteristics and surgical data for each group.

	CON (*N* = 10)	BFR (*N* = 10)	AC (*N* = 10)	One-way ANOVA (*p/*η_p_^2^)
Operated limb	Left: 4/Right: 6	Left: 9/Right: 1	Left: 4/Right: 6	-
Sex	Male: 6/Female: 4	Male: 7/Female: 3	Male: 7/Female: 3	-
Age (yr)	66.3 (7.1)	61.5 (8.8)	64.2 (7.7)	0.410/0.064
Height (cm)	175.4 (8.8)	178.4 (7.2)	175.3 (5.5)	0.565/0.041
Body weight (kg)	90.3 (17.5)	85.3 (15.3)	85.1 (16.5)	0.713/0.025
Blood pressure (mmHg)				
Systolic	129.9 (10.1)	127.5 (5.9)	124.7 (7.9)	0.629/0.034
Diastolic	81.7 (6.9)	81.5 (7.1)	80.8 (4.4)	0.448/0.058
Rest heart rate (bpm)	73.7 (7.2)	66.7 (6.9)	69.8 (7.6)	0.114/0.148
Duration of surgery (min)	111.7 (14.4)	112.3 (14.4)	109.6 (16.4)	0.916/0.006
Blood loss during surgery (mL)	174.0 (63.5)	185.0 (65.4)	168.0 (55.7)	0.824/0.014

Data are provided as mean (standard deviation). CON, control group; BFR, BFR-training group; AC, active control group.

### Exercise Intensity and Physiological Responses During Prehabilitation

Table 2 summarizes the training data during phase 1 (session 1–6) and 2 (session 7–12) of the prehabilitation including exercise intensity, mean HR and RR during training and individual LOP measured before training. All patients in the training groups completed all planned training sessions during the 6-weeks prehabilitation period. The current study documented a dropout rate of 0% and no exercise or BFR-related adverse events. For exercise intensity, there were a significant time × group interaction (*p* < 0.001, η_p_^2^ = 0.599) and time effect (BFR-group: *p* < 0.001, η_p_^2^ = 0.902; AC-group: *p* < 0.001, η_p_^2^ = 0.900). *Post hoc*-tests revealed that the training intensity in both training groups significantly increased during the 2. phase of prehabilitation. No statistically noticeable changes in physiological measures during the training period were detected in any group (Table 2).

**TABLE 2:** Measures related to training during the 6-weeks prehabilitation period of both training groups.

	BFR (*N* = 10)	AC (*N* = 10)	One-way ANOVA/rANOVA (*p/*η_p_^2^)				
			Time		Group		Time x group
			BFR	AC	Phase 1	Phase 2	
Exercise intensity (W)							
Phase 1 (session 1–6)	62.5 (17.5)	69.0 (17.5)	<0.001/0.902	<0.001/0.900	0.417/0.037	0.474/0.029	<0.001/0.599
Phase 2 (session 7–12)	81.8 (15.2)^*^	76.5 (15.2)^*^					
Mean heart rate during training (bpm)							
Phase 1 (session 1–6)	106 (10)	103 (10)	0.542/0.021		0.350/0.049		0.452/0.032
Phase 2 (session 7–12)	108 (11)	103 (11)					
Mean blood pressure during training (mmHg)							
Systolic							
Phase 1 (session 1–6)	143.0 (9.7)	135.9 (9.7)	0.015/0.288		0.053/0.192		0.209/0.086
Phase 2 (session 7–12)	147.2 (9.7)	137.4 (9.7)					
Diastolic							
Phase 1 (session 1–6)	82.5 (7.8)	81.4 (7.8)	0.040/0.214		0.723/0.007		0.890/0.001
Phase 2 (session 7–12)	84.3 (7.9)	83.1 (7.9)					
LOP-Left (mmHg)							
Phase 1 (session 1–6)	226 (14)	228 (14)	0.072/0.169		0.246/0.074		0.100/0.143
Phase 2 (session 7–12)	217 (11)	228 (11)					
LOP-Right (mmHg)							
Phase 1 (session 1–6)	228 (13)	229 (13)	0.024/0.254		0.389/0.042		0.075/0.166
Phase 2 (session 7–12)	220 (14)	228 (14)					

Data are provided as mean (standard deviation). BFR, BFR-training group; AC, active control group; LOP, individual limb occlusion pressure; rANOVA, repeated-measures analysis of variance. *p < 0.05, significantly different to phase 1

### Estimation for Skeletal Muscle Mass of the Lower Extremities

Table 3 summarizes the femoral- and calf-circumference of both operated- (OP) and non-operated (NonOP) legs and their percent difference during the prehabilitation- and post-operative-period. Significant time × group interaction effects were indicated for all measured femoral circumference values (*p* < 0.001, 0.350 < ηp2 < 0.494). Further analyses revealed significant time effects in the BFR-group (*p* < 0.001, 0.674 < η_p_^2^ < 0.754) and CON (*p* ≤ 0.001, 0.627 < η_p_^2^ < 0.728). The AC-group did not indicate any time effects (*p* ≥ 0.078, 0.178 < η_p_^2^ < 0.262). For the calf circumference, no changes were detected in any group despite of the significant interaction effect (*p* = 0.018, η_p_^2^ = 0.205) and main time effect in CON for the NonOP leg (*p* = 0.013, η_p_^2^ = 0.426). Post hoc-tests revealed that the femoral circumference of both legs significantly increased in the BFR-group already at 3w-Prehab (*p* = 0.002). In addition, the BFR-group showed a further improvement in the femoral circumference of the OP leg after the prehabilitation period (*p* = 0.006). The CON did not indicate any changes in the femoral circumference during and after the prehabilitation period (*p* = 0.131). At 3m-Post-OP, both CON and BFR-group showed significantly decreased femoral circumference of both legs compared to the Pre-OP-level (*p* ≤ 0.017), but the BFR-group still remained above the baseline level (*p* = 0.023). At 6m-Post-OP, all femoral circumference in the BFR-group increased again (*p* ≤ 0.030) with significantly higher values compared to the baseline level (*p* < 0.001). In contrast, the CON demonstrated significantly reduced femoral circumference of both legs at 6m-Post-OP with significantly lower values compared to the pre-operative level (*p* ≤ 0.05).

**TABLE 3:** Measures related to skeletal muscle mass of the lower extremities during the prehabilitation- and post-operative period.

	CON (*N* = 10)	BFR (*N* = 10)	AC (*N* = 10)	One-way ANOVA/rANOVA (*p/η*_*p*_^*2*^)								
				Time			Group					Time x group
				CON	BFR	AC	Baseline	3w-Prehab	Pre-OP	3m-Post-OP	6m-Post-OP	
Femoral circumference OP (cm)												
Baseline	58.2 (7.4)	53.2 (4.2)	52.0 (7.3)	<0.001/0.728	<0.001/0.754	0.078/0.266	0.092/0.162	0.224/0.105	0.331/0.079	0.524/0.047	0.592/0.038	<0.001/0.494
3w-Prehab	57.7 (7.5)	55.6 (4.6)^a^	52.5 (7.2)									
Pre-OP	57.6 (7.6)	57.0 (5.2) ^ab^	53.4 (7.1)									
3m-Post-OP	55.9 (7.1) ^abc^	55.0 (3.9) ^ac^	52.8 (6.9)									
6m-Post-OP	55.5 (7.6) ^abc^	56.6 (4.0) ^ad^	53.7 (6.4)									
Femoral circumference NonOP (cm)												
Baseline	58.5 (7.2)	55.0 (4.0)	53.6 (7.3)	0.001/0.627	<0.001/0.674	0.192/0.178	0.229/0.103	0.311/0.083	0.365/0.072	0.502/0.050	0.510/0.049	<0.001/0.350
3w-Prehab	58.5 (7.2)	56.8 (4.4)^a^	54.0 (7.4)									
Pre-OP	58.5 (7.2)	57.6 (4.5)^a^	54.5 (7.3)									
3m-Post-OP	57.2 (7.2) ^abc^	56.9 (4.3)^a^	54.2 (6.7)									
6m-Post-OP	57.4 (7.2) ^abc^	58.0 (4.3) ^ad^	55.0 (6.7)									
%Difference in femoral circumference NonOP - OP												
Baseline	-0.49 (3.24)	-3.44 (1.48)^e^	-3.09 (2.85)	0.002/0.469	0.059/0.254	0.271/0.135	0.037/0.217	0.523/0.047	0.802/0.016	0.748/0.021	0.654/0.031	0.014/0.186
3w-Prehab	-1.43 (3.54)	-2.29 (0.94)	-2.86 (3.16)									
Pre-OP	-1.64 (3.73)	-1.15 (2.74)	-2.11 (3.17)									
3m-Post-OP	-2.27 (4.18)	-3.44 (2.71)	-2.67 (3.44)									
6m-Post-OP	-3.44 (3.50)^a^	-2.54 (2.61)	-2.24 (2.80)									
Calf circumference OP (cm)												
Baseline	39.2 (3.5)	38.3 (3.9)	36.7 (2.7)	0.682/0.014			0.054/0.201					0.412/0.072
3w-Prehab	39.2 (3.5)	38.8 (3.6)	36.8 (2.7)									
Pre-OP	39.2 (3.5)	38.8 (3.7)	37.0 (2.7)									
3m-Post-OP	38.7 (3.8)	38.9 (3.8)	36.7 (2.7)									
6m-Post-OP	38.9 (4.0)	39.1 (3.4)	37.1 (2.7)									
Calf circumference NonOP (cm)												
Baseline	40.2 (3.5)	39.2 (3.4)	37.9 (3.4)	0.013/0.426	0.502/0.053	0.202/0.169	0.333/0.078	0.366/0.072	0.476/0.054	0.549/0.043	0.524/0.047	0.018/0.205
3w-Prehab	40.2 (3.5)	39.3 (3.4)	38.0 (3.4)									
Pre-OP	40.2 (3.5)	39.4 (3.5)	38.3 (3.4)									
3m-Post-OP	39.3 (3.9)	39.5 (3.5)	37.9 (3.3)									
6m-Post-OP	39.4 (3.8)	39.4 (3.6)	37.8 (3.3)									
%Difference in calf circumference NonOP - OP												
Baseline	-2.63 (3.34)	-2.53 (2.20)	-3.16 (4.46)	0.612/0.019			0.241/0.104					0.406/0.030
3w-Prehab	-2.63 (3.34)	-1.36 (1.42)	-3.20 (3.92)									
Pre-OP	-2.63 (3.34)	-1.54 (2.79)	-3.54 (4.04)									
3m-Post-OP	-1.63 (5.23)	-1.36 (2.84)	-3.02 (4.99)									
6m-Post-OP	-1.55 (2.93)	-0.67 (1.51)	-1.93 (4.22)									

Data are provided as mean (standard deviation). CON, control group; BFR = BFR-training group; AC, active control group; rANOVA, repeated-measures analysis of variance; OP, operated leg; NonOP, non-operated leg.

^a^ *p* < 0.05, significantly different to baseline within the respective group.

^b^ *p* < 0.05, significantly different to 3w-Prehab within the respective group.

^c^ *p* < 0.05, significantly different to Pre-OP within the respective group.

^d^ *p* < 0.05, significantly different to 3m-Post-OP within the respective group.

^e^ *p* < 0.05, significantly different to CON within the respective time point.

Regarding the percent difference between the OP and NonOP leg, significant time × group interaction effects were observed for the femoral circumference (*p* = 0.014, η_p_^2^ = 0.186), while for the calf circumference, there were no significant main or interaction effects (Table 3). Further analyses on percent difference in the femoral circumference revealed a significant time effect only for the CON (*p* = 0.002, η_p_^2^ = 0.469) and a significant group effect at the baseline (*p* = 0.037, η_p_^2^ = 0.217). Despite the absence of the significant time effects, both training groups indicated a decreased percent difference in the femoral circumference between both legs following the prehabilitation, which increased again during the post-operative period but not beyond the baseline level (Table 3). In contrast, the percent difference in the femoral circumference between both legs in the CON continually increased during the postoperative study period. It was higher at 6m-Post-OP than the baseline level (*p* = 0.013).

### Knee Swelling Measurements

The knee circumference measured at three different places of both OP and NonOP legs during the prehabilitation- and postoperative-period are presented in Table 4. Regarding the knee circumference, we found no time × group interaction effects (*p* > 0.059, 0.060 < η_p_^2^ < 0.164) except for the upper knee circumference of the Non-OP leg (*p* = 0.010, η_p_^2^ = 0.213), which indicated no further time or group effects (Table 4).

**TABLE 4:** Measures related to knee swelling during the prehabilitation- and post-operative period.

	CON (*N* = 10)	BFR (*N* = 10)	AC (*N* = 10)	One-way ANOVA/rANOVA (*p/*η_p_^2^)								
				Time			Group					Time x group
				CON	BFR	AC	Baseline	3w-Prehab	Pre-OP	3m-Post-OP	6m-Post-OP	
Upper knee circumference OP (cm)												
Baseline	45.0 (4.5)	44.0 (3.1)	43.9 (4.1)	0.686/0.014			0.323/0.083					0.505/0.060
3w-Prehab	45.0 (4.5)	43.7 (3.4)	43.4 (4.0)									
Pre-OP	45.0 (4.5)	43.7 (3.8)	43.3 (4.2)									
3m-Post-OP	46.0 (5.0)	44.3 (3.3)	43.8 (4.2)									
6m-Post-OP	44.8 (5.1)	43.9 (3.4)	43.6 (4.2)									
Upper knee circumference NonOP (cm)												
Baseline	44.5 (3.8)	43.3 (3.0)	42.2 (3.1)	0.057/0.287	0.069/0.209	0.278/0.129	0.485/0.052	0.509/0.049	0.600/0.037	0.793/0.017	0.805/0.016	0.010/0.213
3w-Prehab	44.5 (3.8)	43.2 (3.3)	42.4 (4.0)									
Pre-OP	44.5 (3.8)	43.8 (3.8)	42.6 (4.2)									
3m-Post-OP	44.0 (5.5)	43.6 (3.1)	42.7 (4.1)									
6m-Post-OP	43.7 (5.3)	43.7 (3.3)	42.6 (4.1)									
%Difference upper knee NonOP - OP												
Baseline	1.22 (2.04)	1.78 (1.81)	3.90 (2.01)^e^	0.017/0.445	0.073/0.239	0.093/0.250	0.012/0.280	0.181/0.119	0.076/0.174	0.049/0.200	0.097/0.159	0.049/0.172
3w-Prehab	1.22 (2.04)	1.62 (1.48)	2.65 (1.63)									
Pre-OP	1.22 (2.04)	0.31 (1.04)	1.96 (1.40)									
3m-Post-OP	4.39 (2.31)^*^	1.77 (1.51)	2.84 (2.79)									
6m-Post-OP	2.64 (1.78)^d^	1.09 (0.74)	2.79 (2.59)									
Middle knee circumference OP (cm)												
Baseline	43.8 (4.5)	42.7 (3.2)	42.7 (3.2)	0.172/0.065			0.096/0.165					0.134/0.123
3w-Prehab	43.8 (4.5)	42.4 (2.5)	42.2 (3.2)									
Pre-OP	43.8 (4.5)	42.4 (2.9)	42.1 (3.3)									
3m-Post-OP	44.8 (4.1)	42.7 (2.7)	42.6 (3.0)									
6m-Post-OP	43.6 (4.0)	41.8 (2.9)	42.4 (2.9)									
Middle knee circumference NonOP (cm)												
Baseline	42.9 (4.5)	41.5 (3.4)	41.0 (3.9)	0.563/0.024			0.264/0.097					0.555/0.058
3w-Prehab	42.9 (4.5)	41.8 (3.6)	41.2 (3.9)									
Pre-OP	42.9 (4.5)	41.7 (3.7)	41.2 (4.1)									
3m-Post-OP	42.7 (5.4)	41.8 (3.2)	41.2 (4.0)									
6m-Post-OP	42.3 (5.2)	41.6 (3.3)	41.1 (4.1)									
%Difference middle knee NonOP - OP												
Baseline	1.87 (1.74)	2.85 (2.24)	4.20 (1.41)	0.140/0.072			0.046/0.211					0.107/0.133
3w-Prehab	1.87 (1.74)	1.54 (2.24)	2.58 (1.78)									
Pre-OP	1.87 (1.74)	1.82 (2.67)	2.05 (1.75)									
3m-Post-OP	4.86 (2.94)	2.27 (1.75)	3.25 (4.00)									
6m-Post-OP	3.14 (2.18)	0.75 (2.65)	3.19 (2.96)									
Lower knee circumference OP (cm)												
Baseline	39.4 (3.5)	38.5 (3.4)	39.0 (3.2)	0.017/0.149			0.067/0.188					0.059/0.164
3w-Prehab	39.4 (3.5)	37.8 (3.1)	38.7 (3.3)									
Pre-OP	39.4 (3.5)	37.7 (3.8)	38.6 (3.3)									
3m-Post-OP	40.3 (4.1)	38.6 (3.4)	38.6 (3.3)									
6m-Post-OP	39.4 (4.0)	38.3 (3.7)	38.8 (3.2)									
Lower knee circumference NonOP (cm)												
Baseline	39.0 (3.6)	37.5 (3.1)	37.5 (3.3)	0.335/0.008			0.335/0.081					0.384/0.076
3w-Prehab	39.0 (3.6)	37.3 (2.8)	37.4 (3.3)									
Pre-OP	39.0 (3.6)	37.3 (3.0)	37.7 (3.3)									
3m-Post-OP	38.8 (4.1)	37.3 (2.7)	37.7 (3.3)									
6m-Post-OP	38.7 (3.9)	37.4 (2.8)	37.7 (3.1)									
%Difference lower knee NonOP - OP												
Baseline	0.76 (3.51)	2.60 (1.89)	3.83 (2.16)	<0.001/0.373			0.573/0.042					0.282/0.091
3w-Prehab	0.76 (3.51)	1.39 (2.00)	3.49 (2.58)									
Pre-OP	0.76 (3.51)	1.04 (1.89)	2.54 (1.78)									
3m-Post-OP	4.06 (2.42)	3.51 (2.65)	2.32 (3.30)									
6m-Post-OP	1.90 (1.55)	2.30 (2.25)	2.94 (2.21)									

Data are provided as mean (standard deviation). CON, control group; BFR, BFR-training group; AC, active control group; rANOVA, repeated-measures analysis of variance; OP, operated leg; NonOP, non-operated leg.

^a^ *p* < 0.05, significantly different to baseline within the respective group.

^b^ *p* < 0.05, significantly different to 3w-Prehab within the respective group.

^c^ *p* < 0.05, significantly different to Pre-OP within the respective group.

^d^ *p* < 0.05, significantly different to 3m-Post-OP within the respective group.

^e^ *p* < 0.05, significantly different to CON within the respective time point.

Further analyses revealed a significant time effect only for the lower knee circumference of the OP leg (*p* = 0.017, η_p_^2^ = 0.072), indicating that knee circumference increased at 3m-Post-OP and returned to baseline level at 6m-Post-OP.

Regarding the knee swelling accessed by the percent difference between OP and NonOP knee, a significant time × group interaction effect was found only for upper knee (*p* = 0.049, η_p_^2^ = 0.172). For the swelling measured at middle knee, we found a significant group effect (*p* = 0.046, η_p_^2^ = 0.211) indicating lower values in the BFR-group compared to other groups. For lower knee, found a significant time effect (*p* < 0.001, η_p_^2^ = 0.373) with a continually decreased swelling during the prehabilitation period, which increased at 3m-Post-OP and returned to baseline level at 6m-Post-OP.

Further analyses for the swelling measured at upper knee detected significant time effect only in the CON (*p* = 0.017, η_p_^2^ = 0.445). In addition, there were significant group effects at baseline (*p* < 0.012, η_p_^2^ = 0.280) and 3m-Post-OP (*p* < 0.049, η_p_^2^ = 0.200). At baseline, the upper knee swelling was significantly higher in the AC-group that the CON (*p* = 0.043), while the BFR-group did not differ to other groups (*p* ≥ 0.093). The CON demonstrated a greater increase in the upper knee swelling with higher level at 3m-Post-OP compared to the BFR-group (*p* = 0.046), which significantly decreased again at 6m-Post-OP (*p* = 0.044). Despite being statistically non-significant, both BFR- and AC-groups demonstrated decreased knee swelling values at 6m-Post-OP compared to the baseline level (Table 4).

### Functionality Measurements

Table 5 summarizes the ROM assessed during active extension and flexion. For all ROM measurements, there were no significant time × group interaction effects (*p* ≥ 0.403, 0.043 < η_p_^2^ < 0.073). Further, significant time effects were detected only for OP leg (extension: *p* = 0.012, η_p_^2^ = 0.138; flexion: *p* < 0.001, η_p_^2^ = 0.468) indicating a continually improved ROM during overall study period. No main group effects were found for all ROM measures (*p* ≥ 0.169, 0.048 < η_p_^2^ < 0.128).

**TABLE 5:** Active range of motion of knee joint during the prehabilitation- and post-operative period.

	CON (*N* = 10)	BFR (*N* = 10)	AC (*N* = 10)	One-way ANOVA/rANOVA (*p/*η^2^_p_)								
				Time			Group					Time x group
				CON	BFR	AC	Baseline	3w-Prehab	Pre-OP	3m-Post-OP	6m-Post-OP	
ROM extension OP (°)												
Baseline	2.60 (1.90)	3.40 (3.20)	2.40 (2.76)	0.012/0.138			0.169/0.128					0.723/0.043
3w-Prehab	2.60 (1.90)	2.70 (2.83)	2.30 (2.83)									
Pre-OP	2.60 (1.90)	2.70 (2.41)	2.30 (2.83)									
3m-Post-OP	3.30 (2.98)	1.50 (3.24)	2.00 (1.63)									
6m-Post-OP	2.00 (2.58)	0.70 (1.57)	1.20 (1.14)									
ROM extension NonOP (°)												
Baseline	3.90 (4.23)	0.30 (0.95)	1.20 (1.75)	0.607/0.022			0.699/0.027					0.580/0.056
3w-Prehab	3.90 (4.23)	0.70 (1.49)	0.90 (1.52)									
Pre-OP	3.90 (4.23)	0.50 (1.08)	1.70 (2.67)									
3m-Post-OP	2.60 (3.57)	1.20 (2.10)	1.30 (1.49)									
6m-Post-OP	1.10 (1.45)	0.20 (0.63)	0.60 (1.07)									
ROM flexion OP (°)												
Baseline	116.5 (14.8)	113.7 (11.5)	113.1 (4.2)	<0.001/0.468			0.513/0.048					0.403/0.073
3w-Prehab	116.4 (14.3)	116.2 (10.8)	112.7 (3.5)									
Pre-OP	117.0 (14.4)	116.0 (10.7)	112.4 (3.1)									
3m-Post-OP	114.7 (7.1)	117.9 (6.0)	113.3 (10.1)									
6m-Post-OP	122.0 (8.5)	119.5 (6.6)	115.3 (7.9)									
ROM flexion NonOP (°)												
Baseline	127.3 (9.8)	128.1 (9.1)	126.4 (7.7)	0.337/0.043			0.342/0.082					0.820/0.033
3w-Prehab	127.3 (9.8)	130.3 (8.5)	127.7 (6.9)									
Pre-OP	127.3 (9.8)	128.5 (8.6)	126.4 (8.0)									
3m-Post-OP	126.8 (9.5)	130.5 (10.5)	126.6 (9.2)									
6m-Post-OP	127.3 (10.1)	128.5 (8.6)	126.6 (9.2)									

Data are provided as mean (standard deviation). CON, control group; BFR, BFR-training group; AC, active control group; rANOVA, repeated-measures analysis of variance; OP, operated leg; NonOP, non-operated leg.

^a^ *p* < 0.05, significantly different to baseline within the respective group.

^b^ *p* < 0.05, significantly different to 3w-Prehab within the respective group.

^c^ *p* < 0.05, significantly different to CON within respective the time point.

**FIGURE 2:**
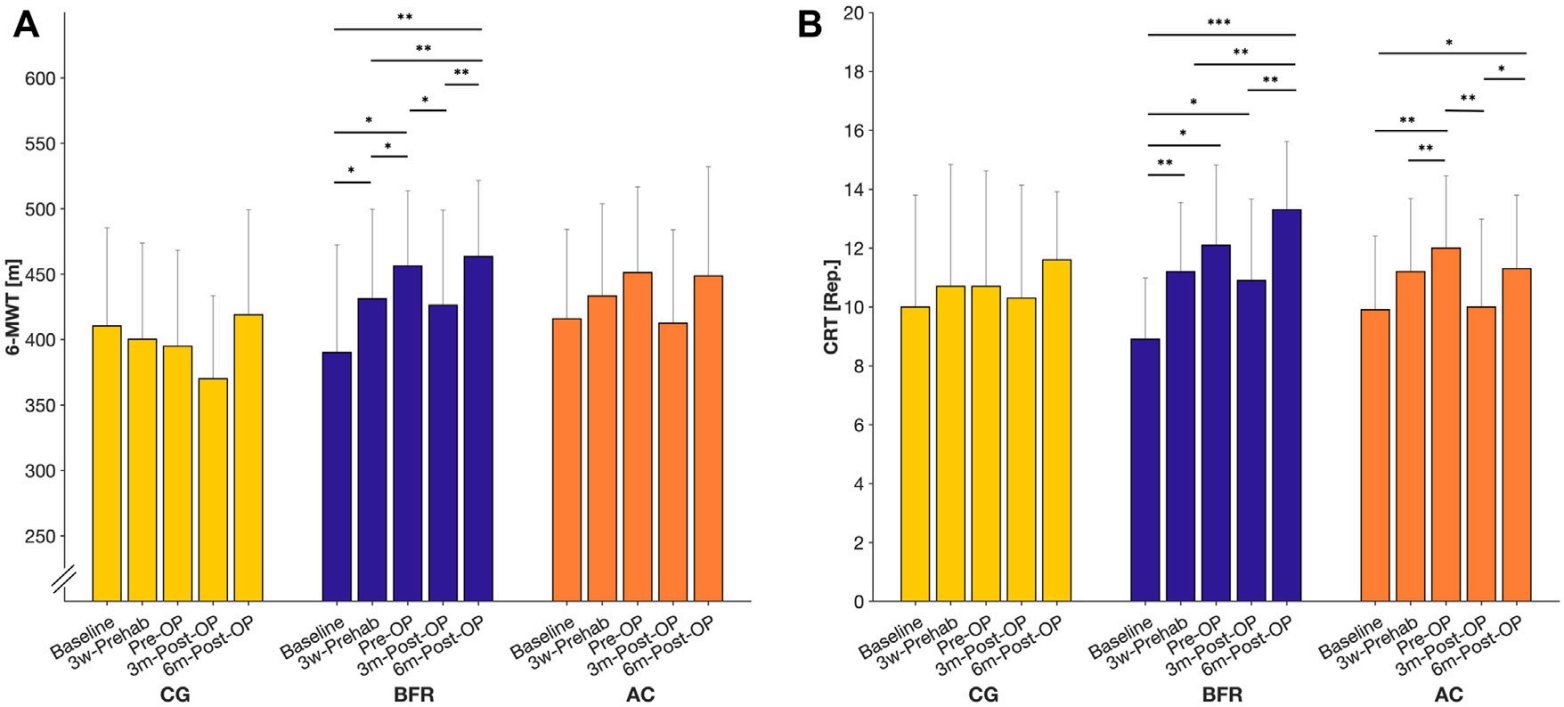
Measures related to the 6-min walking test (6-MWT; **(A)** and chair-rising test (CRT; **(B)** during prehabilitation- and post-operative period. Data are provided as mean (standard deviation). CON = control group; BFR = BFR-training group; AC = active control group. ***p < 0.001, **p < 0.01, *p < 0.05, significant difference within the respective group.

For 6-MWT (Figure 2A), we found a significant time × group interaction effect (*p* = 0.012, η_p_^2^ = 0.209). Further analyses revealed a significant time effect only in BFR-group (*p* < 0.001, η_p_^2^ = 0.677). *Post hoc*-tests for BFR-group indicated a significant improvement in 6-MWT already at 3w-Prehab compared to baseline (390 ± 82 m to 431 ± 69 m, *p* = 0.034), which increased further after the prehabilitation (to 456 ± 58 m) with a significantly higher level to the baseline- and 3w-Prehab-level (*p* ≤ 0.048). At 3m-Post-OP, the BFR-group showed a significant deterioration in 6-MWT compared to Pre-OP (to 426 ± 73 m, *p* = 0.05), but it pronounced recuperated at 6m-Post-OP (to 464 ± 58 m, *p* = 0.002). Consequently, the BFR-group demonstrated a significantly higher ability in 6-MWT at 6m-Post-OP compared to baseline- (*p* = 0.004) and 3w-Prehab-level (*p* = 0.007).

Regarding the CRT (Figure 2B), there was a significant time x group effect (*p* = 0.007, η_p_^2^ = 0.205). In addition, we found significant time effects in BFR- (*p* < 0.001, η_p_^2^ = 0.671) and AC-group (*p* < 0.001, η_p_^2^ = 0.596), whereas no changes occurred in the CON. According to *Post hoc*-tests, the patients in the BFR-group significantly improved in the CRT already at 3w-Prehab compared to the baseline (8.90 ± 2.08 reps. to 11.20 ± 2.35 reps., *p* = 0.012). In comparison, the AC-group showed a significant improvement only after the prehabilitation period (9.90 ± 2.51 reps. to 12.00 ± 2.49 reps., *p* = 0.006). At 3m-Post-OP, the AC-group exhibited a significant deterioration in the CRT compared to Pre-OP (to 10.00 ± 2.98 reps., *p* = 0.002), which pronounced improved at 6m-Post-OP again (to 11.30 ± 2.50 reps., *p* = 0.019), and was significantly higher to baseline level (*p* = 0.026). In contrast, the BFR-group indicated no statistically significant change in the CRT at 3m-Post-OP (12.10 ± 2.73 reps. to 10.90 ± 2.77 reps., *p* = 1.00), which was still higher compared to the baseline level (*p* = 0.038). At 6m-Post-OP, the BFR-group improved again (to13.30 ± 2.31 reps., *p* = 0.003) remaining above the baseline- and 3w-Prehab level (*p* ≤ 0.004).

### Muscular Strength of the Lower Extremities

The results regarding the muscular strength of both OP and NonOP legs and their percent difference during the prehabilitation and post-operative period are presented in Table 6. Significant interaction effects were observed for all measured muscle strength indices (*p* < 0.001, 0.567 < η_p_^2^ < 0.625). Further analyses revealed a significant main time effect for all muscle strength measures in all groups (*p* < 0.05, 0.298 < η_p_^2^ < 0.916). In addition, there were significant group effects for all measured leg strength indices (*p* ≤ 0.046, 0.204 < η_p_^2^ < 0.676) excepting for leg extension of OP leg at baseline (*p* = 0.077, η_p_^2^ = 0.173) and at 3w-Prehab (*p* = 0.230, η_p_^2^ = 0.103). *Post hoc*-tests revealed that both training groups significantly improved in all measured leg strength indices already at 3w-Prehab (BFR-group: *p* ≤ 0.01; AC-group: *p* ≤ 0.026). At Pre-OP, the BFR-group indicated more pronounced improvements in all leg strength measures (i.e., 3w-Prehab to Pre-OP: *p* < 0.001) with significantly higher values compared to other groups (*p* < 0.05). No changes occurred in the CON during the prehabilitation period (*p* = 0.100). At 3m-Post-OP, significant reductions in leg strength measures were observed in BFR-group (*p* ≤ 0.01) except for the leg extension of the NonOP leg (*p* = 0.308), which were still above the baseline level (*p* ≤ 0.01). The AC-group indicated significantly decreased muscular strength in both leg-extension (only in NonOP leg) and -curl (in both legs) at 3m-Post-OP even to the baseline level (*p* ≤ 0.05). Similarly, the patients in the CON showed a significant decrement in leg extension of both OP and NonOP legs at 3m-Post-OP with a lower value compared to the pre-operative level (*p* ≤ 0.031). At the same time, no changes occurred in leg curl (*p* ≥ 0.187). At 6m-Post-OP, both training groups significantly improved again in all leg strength measures (BFR-group: *p* ≤ 0.029; AC-group: *p* ≤ 0.031) with a significant difference to baseline- (BFR-group: *p* < 0.001 for all measures; AC-group: *p* ≤ 0.029 only for leg curl of both legs) and to 3w-Prehab-level (only in BFR-group: *p* ≤ 0.038 for all measures). The CON also indicated significant improvements but only in muscular strength of OP leg (leg extension: *p* = 0.005; leg curl: *p* = 0.007). Consequently, there were significant differences in leg strength between BFR-group and other groups during the overall post-operative period (AC-group: *p* ≤ 0.032; CON: *p* ≤ 0.020) excepting for the leg extension of OP leg at 3m-post-OP between BFR-group and CON (*p* = 0.265).

**TABLE 6:** Measures related to muscular strength of lower extremities during the prehabilitation- and post-operative period.

				One-way ANOVA/rANOVA (*p*/η^2^_p_)								
	CON (*N* = 10)	BFR (*N* = 10)	AC (*N* = 10)	Time					Group			Time x group
				CON	BFR	AC	Baseline	3w-Prehab	Pre-OP	3m-Post-OP	6m-Post-OP	
Leg extension OP (kg)												
Baseline	20.3 (8.1)	13.8 (9.1)	11.8 (7.8)	0.001/0.591	<0.001/0.863	0.014/0.392	0.077/0.173	0.230/0.103	0.003/0.351	0.033/0.223	0.002/0.364	<0.001/0.614
3w-Prehab	20.3 (7.1)	21.9 (9.1)a	15.3 (9.8)a									
Pre-OP	20.3 (7.1)^f^	31.5 (10.4) ^ab^	16.0 (10.2)^a^f									
3m-Post-OP	15.0 (6.3) ^abc^	22.1 (10.4) ^ac^	11.1 (9.5)f									
6m-Post-OP	18.5 (7.0)^d^f	30.8 (11.1) ^abd^	15.3 (9.4)^d^f									
Leg extension NonOP (kg)												
Leg extension NonOP (kg)				0.018/0.404	<0.001/0.851	0.003/0.503	0.026/0.237	0.021/0.250	0.003/0.348	0.002/0.379	0.000/0.445	<0.001/0.587
Baseline	28.3 (10.4)	24.3 (7.3)	17.5 (7.1)e									
3w-Prehab	28.5 (10.5)	31.8 (7.6)a	20.3 (8.1)^a^f									
Pre-OP	28.3 (10.7)f	38.4 (9.7) ^ab^	22.0 (8.7)^a^f									
3m-Post-OP	22.5 (8.6) ^abc^f	34.1 (8.5)a	18.8 (9.2)^c^f									
6m-Post-OP	27.0 (9.5)f	39.6 (9.7) ^abd^	20.8 (8.3)^d^f									
%Difference leg extension NonOP - OP												
Baseline	-34.4 (36.0)	-64.6 (31.9)	-51.0 (31.3)	0.367/0.038						0.003/0.366		0.063/0.150
3w-Prehab	-33.1 (26.2)	-40.3 (23.2)a	-39.6 (35.9)									
Pre-OP	-31.9 (28.5)	-21.0 (16.2) ^ab^	-44.1 (42.5)									
3m-Post-OP	-39.5 (34.0)	-46.7 (29.2)c	-69.1 (40.7)									
6m-Post-OP	-37.0 (28.4)	-27.3 (23.9) ^abd^	-38.1 (26.8)									
Leg curl OP (kg)												
Baseline	10.8 (3.1)	9.0 (4.6)	6.4 (3.4)e	0.038/0.334	<0.001/0.916	0.003/0.502	0.046/0.204	0.017/0.260	<0.001/0.557	0.001/0.416	<0.001/0.645	<0.001/0.625
3w-Prehab	10.8 (3.1)	14.2 (4.6)a	9.0 (3.6)^a^f									
Pre-OP	11.0 (2.9)f	19.1 (4.8) ^ab^	9.8 (3.6)^a^f									
3m-Post-OP	8.8 (2.7)f	14.6 (5.3) ^ac^	6.4 (4.6)^c^f									
6m-Post-OP	11.0 (1.7)^d^f	19.6 (4.1) ^abd^	9.0 (4.3) ^ad^f									
Leg curl NonOP (kg)												
Baseline	13.3 (4.6)	14.6 (3.9)	10.0 (3.1)f	0.049/0.298	<0.001/0.815	<0.001/0.617	0.039/0.213	0.011/0.286	<0.001/0.550	<0.001/0.597	<0.001/0.676	<0.001/0.567
3w-Prehab	13.8 (4.3)f	19.0 (4.3)a	13.3 (4.4)^a^f									
Pre-OP	13.5 (4.3)f	23.1 (3.7) ^ab^	14.5 (4.2)^a^f									
3m-Post-OP	11.3 (2.7)f	19.5 (3.1) ^ac^	10.8 (4.4) ^bc^f									
6m-Post-OP	12.8 (4.0)f	22.6 (2.6) ^abd^	12.5 (3.5) ^ad^f									
%Difference leg curl NonOP - OP												
Baseline	-19.8 (25.97)	-54.5 (35.5)	-54.4 (40.3)	0.105/0.081						<0.001/0.481		0.100/0.134
3w-Prehab	-24.5 (18.5)	-30.8 (27.5)	-39.9 (27.0)									
Pre-OP	-19.8 (18.9)	-20.4 (17.3)	-40.9 (23.0)									
3m-Post-OP	-26.5 (19.3)	-33.0 (29.5)	-67.5 (38.4)									
6m-Post-OP	-12.0 (19.9)	-15.8 (18.7)	-40.2 (28.5)									

Data are provided as mean (standard deviation). CON, control group; BFR, BFR-training group; AC, active control group; rANOVA, repeated-measures analysis of variance; OP, operated leg; NonOP, non-operated leg.

^a^ *p* < 0.05, significantly different to baseline within the respective group.

^b^ *p* < 0.05, significantly different to 3w-Prehab within the respective group.

^c^ *p* < 0.05, significantly different to Pre-OP within the respective group.

^d^ *p* < 0.05, significantly different to 3m-Post-OP within the respective group.

^e^ *p* < 0.05, significantly different to CON within the respective time point.

^f^ *p* < 0.05, significantly different to BFR-group within the respective time point.

Regarding the strength deficit of the OP leg accessed by the percent difference between OP and Non-OP leg during leg-extension and -curl, we found no significant interaction (*p* ≥ 0.063, 0.134 < η_p_^2^ < 0.150) and time effects (*p* ≥ 0.105, 0.038 < η_p_^2^ < 0.081). There were significant main group effects (*p* ≤ 0.003, 0.366 < η_p_^2^ < 0.481) with lower values in BFR-group compared to other groups.

### Subjective Surveys and Questionnaires

**FIGURE 3:**
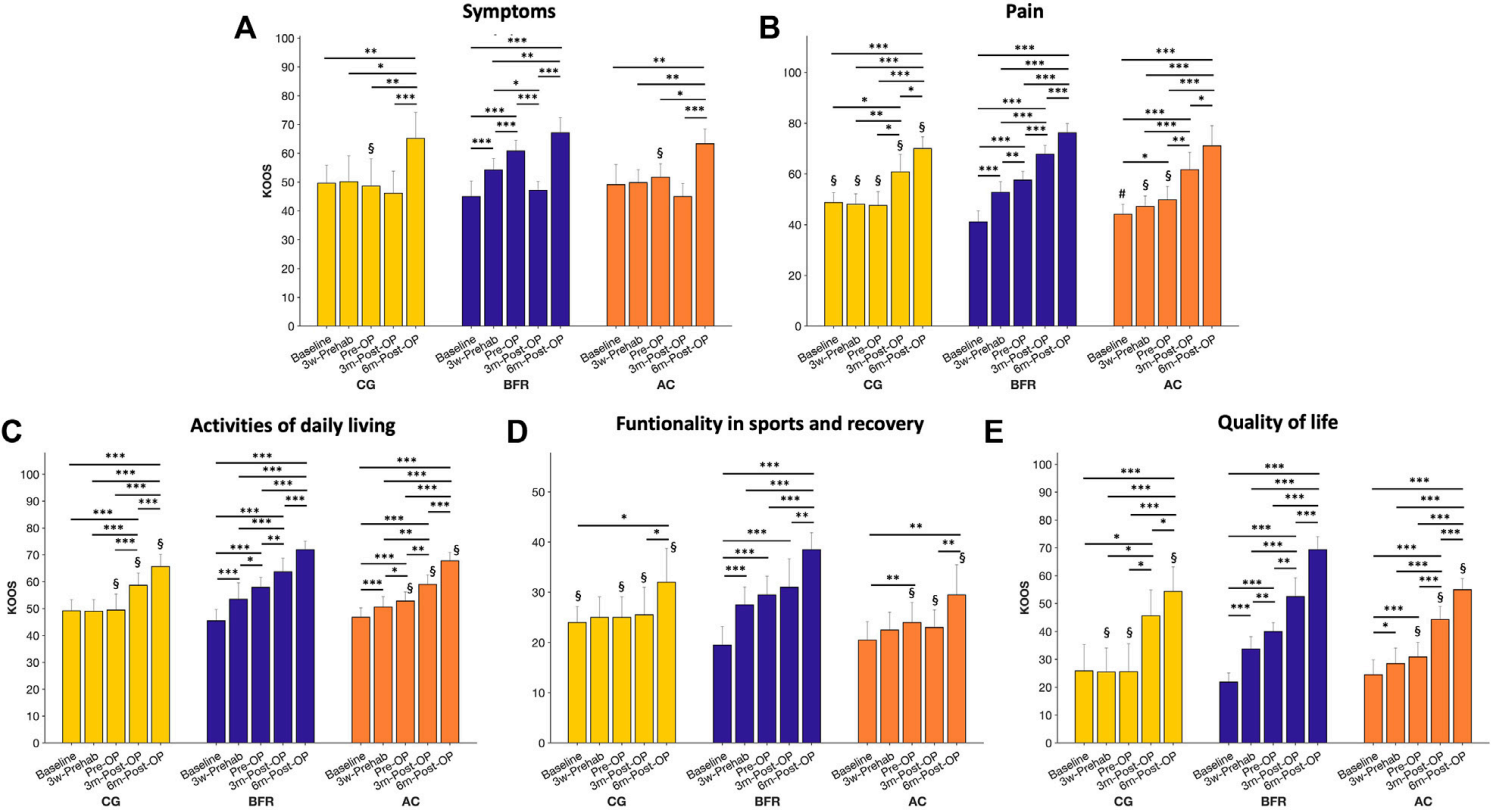
Presentation of the five subparameters (Symptoms, Pain, Activities of daily living, Functionality in sports and recovery, Quality of life) of the Knee Injury and Osteoarthritis Outcome Score (KOOS) during prehabilitation- and post-operative period. Data are provided as mean (standard deviation). CON, control group; BFR, BFR-training group; AC, active control group. ***p < 0.001, **p < 0.01, *p < 0.05, significant difference within the respective group. ^§^ *p* < 0.05, significantly different to BFR-group within the respective timepoint. ^#^ *p* < 0.05, significantly different to CON within the respective timepoint.

The analysis on the KOOS (Figure 3) demonstrated significant time × group interaction effects for all evaluated dimensions (*p* ≤ 0.004, 0.268 < η_p_^2^ < 0.416). Further analyses revealed a significant main time effect for all measures of KOOS in all groups (CON: *p* ≤ 0.004, 0.475 < η_p_^2^ < 0.907; BFR-group: *p* < 0.001, 0.869 < η_p_^2^ < 0.978; AC-group: *p* ≤ 0.001, 0.571 < η_p_^2^ < 0.951). In addition, there were significant group effects for all measures of KOOS (*p* ≤ 0.049, 0.200 < η_p_^2^ < 0.581) excepting for the dimension *symptoms* at baseline, 3w-Prehab, 3m-Post-OP, and 6m-Post-OP (*p* ≥ 0.207, 0.029 < η_p_^2^ < 0.110) and *quality of life-related to the affected knee* at baseline (*p* = 0.398, η_p_^2^ = 0.066).

*Post hoc*-tests for the KOOS related to *symptoms* (Figure 3A) revealed a significant improvement only in the BFR-group during (45.0 ± 5.4 to 54.2 ± 3.9, *p* < 0.001) and after the prehabilitation period (to 60.8 ± 3.7, *p* < 0.001) with a significant higher value compared to other groups at Pre-OP (CON: 51.7 ± 4.7; AC-group: 48.6 ± 9.5, *p* ≤ 0.01). No difference was observed between CON and AC-group (*p* = 0.893). At 3m-Post-OP, the BFR-group indicated a significant lower KOOS related to *symptoms* (47.2 ± 3.0) compared to 3w-Prehab- (*p* = 0.021) and Pre-OP-level (*p* < 0.001), but still similar level to other groups (CON: 45.0 ± 4.5; AC-group: 46.1 ± 7.7). At 6m-Post-OP, the KOOS related to *symptoms* increased in all groups (CON: 63.4 ± 5.1; BFR-group: 67.1 ± 3.6; AC-group: 65.2 ± 9.0) with a significantly higher value compared to previous level (*p* ≤ 0.05).

The KOOS related to *pain* (Figure 3B) in the CON was significantly higher at baseline compared to other groups (CON: 48.7 ± 3.9; BFR-group: 41.1 ± 4.3; AC-group: 44.2 ± 3.8, *p* ≤ 0.048), but there was no difference between both training groups (*p* = 0.299). The BFR-group significantly improved the KOOS related to *pain* during (to 52.8 ± 4.1, *p* < 0.001) and after the prehabilitation period (to 57.6 ± 3.4, *p* < 0.001), while no changes occurred in the CON (at 3w-Prehab: 48.0 ± 4.1; at Pre-OP: 47.6 ± 5.4, *p* = 1.00). The AC-group showed an improvement in the KOOS related to *pain* only after the prehabilitation (i.e., at Pre-OP to 49.8 ± 5.2) with a significant difference to baseline (*p* = 0.033). Consequently, the BFR-group exhibited significant higher KOOS related to *pain* compared to other groups both at 3w-Prehab (*p* ≤ 0.048) and at Pre-OP (*p* ≤ 0.003). During the post-operative period, all groups indicated further improvements in the KOOS related to *pain* with a significant higher value to all pre-operative time points (at 3m-Post-OP: 60.8 ± 6.9 vs. 67.8 ± 3.5 vs. 61.7 ± 6.8; at 6m-Post-OP: 70.0 ± 4.7 vs. 76.2 ± 3.6 vs. 71.1 ± 7.9 in CON, BFR-, AC-group, respectively). In addition, the BFR-group indicated a significant higher KOOS related to *pain* during the post-operative period compared to the CON (*p* ≤ 0.050 at both 3m- and 6m-Post-OP), whereas the AC-group did not differ to any other groups (*p* ≥ 0.10).

The analysis on the KOOS related to the *activities of daily living* (Figure 3C) revealed a continuous improvement in both training groups during the overall study period (BFR-group: 45.5 ± 4.2 to 53.5 ± 6.1 to 57.9 ± 3.7 to 63.7 ± 5.1 to 71.9 ± 3.1; AC-group: 46.7 ± 3.5 to 50.5 ± 3.8 to 52.8 ± 3.4 to 59.0 ± 3.5 to 67.8 ± 3.1; *p* ≤ 0.047), while the CON showed a significant improvement only during the post-operative period (49.2 ± 4.2 to 49.0 ± 4.4 to 49.4 ± 6.0 to 58.7 ± 4.5 to 65.6 ± 4.6; *p* < 0.001). Moreover, the BFR-group indicated significant higher KOOS related to activities of daily living compared to other groups at Pre-OP (*p* ≤ 0.05), 3m- (*p* ≤ 0.05), and 6m-Post-OP (*p* ≤ 0.05), whereas no differences were observed between CON and AC-group (*p* ≥ 0.325).

Regarding the functionality in sports and recovery (Figure 3D), the CON showed a higher KOOS compared to BFR-group at the baseline (24.0 ± 3.2 vs. 19.5 ± 3.7; *p* = 0.024), but the AC-group indicated no difference to any other groups (20.5 ± 3.7; *p* ≥ 0.105). Only in the BFR-group, the functionality in sports and recovery already improved at 3w- Prehab (to 27.5 ± 3.5; *p* = 0.001) with a significant higher value compared to AC-group (22.5 ± 3.5; *p* = 0.024). At the Pre-OP, both training groups demonstrated higher sports and recovery functionality than the baseline level (BFR-group: to 29.5 ± 3.7; AC-group: to 24.0 ± 3.9; *p* ≤ 0.013). No changes occurred in the CON during (to 25.0 ± 4.1) and after (to 25.0 ± 4.1) the prehabilitation period. At the 3m-Post-OP, only in the BFR-group, the functionality in sports and recovery was higher compared to the baseline level (to 31.0 ± 5.7; *p* < 0.001). At the 6m-Post-OP, the functionality in sports and recovery in the BFR-group was significant higher compared to each of all other time points (38.5 ± 3.4; *p* ≤ 0.003), whereas the CON (32.0 ± 6.7) and AC-group (29.5 ± 6.0) demonstrated a significant higher value only compared to the baseline- (*p* ≤ 0.031) and 3m-Post-OP-level (*p* ≤ 0.037). After the Pre-OP until 6m-Post-OP, the functionality in sports and recovery was higher in the BFR-group compared to other group (*p* ≤ 0.05).

The KOOS related to the quality of life-related to the affected knee (Figure 3E) increased in both training groups already at 3w-Prehab (BFR-group: 21.9 ± 3.3 to 33.8 ± 4.4; AC-group: 24.5 ± 5.3 to 28.5 ± 5.5; *p* ≤ 0.017), and thus the BFR-group indicated higher value compared to the CON (25.5 ± 8.6; *p* = 0.024). At Pre-OP, the BFR-group demonstrated a more pronounced improvement in the quality of life-related to the affected knee (to 40.0 ± 3.2; *p* = 0.011) with a higher value compared to other groups (CON: to 25.6 ± 10.0; AC-group: 30.9 ± 5.2; *p* ≤ 0.017). During the post-operative period, all groups demonstrated an increased quality of life-related to the affected knee compared to each of other previous time points (CON: to 45.6 ± 9.2 to 54.4 ± 8.99; BFR-group: to 52.5 ± 6.8 to 69.4 ± 4.6; AC-group: to 44.4 ± 4.6 to 55.0 ± 4.0; *p* ≤ 0.032), whereas the BFR-group still exhibited higher values compared to other groups (*p* ≤ 0.05) except for 3m-Post-OP in the CON (*p* = 0.119).

## Discussion

The present study aimed to investigate the impact of a 6-week prehabilitation with BFRE on skeletal muscle mass and strength before and after elective primary TKA. The main findings were, that BFR prehabilitation can reduce perceived pain and increase muscle mass and strength significantly more than prehabilitation without BFR before elective TKA surgery. Furthermore, BFR prehabilitation shows a positive influence on postoperative regeneration of skeletal muscle mass, strength and functionality compared to AC and CON, with supportive effects on subjective pain perception and QoL as well.

The present findings at baseline show that muscle mass and strength of patients receiving primary TKA is highly affected by OA. In addition to the subjectively perceived and objectively measurable decrease in functionality, the difference between the patients’ extremities is particularly remarkable. Our data show significant differences between the muscle mass as well as muscle strength between the OP and NonOP leg at baseline (Table 3, 6). These results are of particular significance, since it is known that the most important predictive parameters concerning a successful rehabilitation after surgery are preoperative strength, ROM, perceived pain and the ability to complete functional tasks (Topp et al., 2009). This condition is expected to be caused by preoperative immobility and OA-induced arthrogenic muscle inhibition (Mayer et al., 2017). Since comparative literature show similar preoperative deficits (Ikeda et al., 2005; Dreyer et al., 2013), this circumstance could contribute to the dissatisfaction rate of approximately 20% after primary TKA (Bourne et al., 2010; Canovas and Dagneaux, 2018). Therefore, preoperative modification of physical capacities could be a tool to increase rehabilitation success and satisfaction after TKA.

Several studies supported preoperative well-being and postoperative rehabilitation through prehabilitation (Walls et al., 2010; Shaarani et al., 2013; Calatayud et al., 2017). However, current meta-analyses show only a slight to moderate influence of previous prehabilitation concepts on pre- and postoperative clinical outcomes (Wang et al., 2016; Moyer et al., 2017). These results are essentially influenced by the fact that existing methods of exercise led to increased pain, have been simplified and thus do no longer provide the necessary stimulus for muscle development. BFR training avoids this problem by using metabolic rather than mechanical stimuli to increase muscle mass and strength.

The present study suggests that prehabilitation with a 6-week cycling ergometer protocol is sufficient to enhance muscle mass, strength and subjective pain significantly before surgery. In comparison, BFRE was able to increase muscle mass already after 3 weeks of prehabilitation (Table 3), enhance muscle strength (Table 6) and functional performance (Figure 1) superior to AC. These findings are well in line with the literature, illustrating that a 6-week knee extensor-based prehabilitation protocol with BFR induce significant improvements in muscle mass and strength in patients receiving ACL-reconstruction (Kacin et al., 2021). Even though only an indirect measurement tool was chosen to represent muscle mass in the present study, these results allow the interpretation that changes in leg circumference are primarily explained by muscular gains. Furthermore, comparison between the OP and NonOP legs revealed, that BFR-prehabilitation can address successfully preoperative muscular disbalances (Table 3, 6). In addition to the choice of exercise technique, the duration of the prehabilitation is also very important. In a study by Grapar Zargi and colleagues (Grapar Zargi et al., 2016), five times of BFRE within 10 days before an elective ACL reconstruction could not show any influence on the muscle mass and muscle strength. Considering the present results, a prehabilitation duration between three and 6 weeks with strength or endurance BFRE seems to be able to induce significant muscular effects before an elective joint surgery.

The improvements in muscle mass and strength of the BFR-group during and after the prehabilitation phase were associated with an equal enhancement in all five different subparameters of the KOOS score (Figure 3). These findings are well in line with previous literature reporting the positive influence of increased muscle mass and strength on subjective pain perception and QoL in OA-affected patients (Davison et al., 2017; Kemnitz et al., 2017). A meta-analysis by Ferlito et al. (2020)) was able to show that BFRE leads to similar gains in muscle mass and strength with concurrent reductions in perceived pain like high-intensity training. Although there is no high-intensity comparison group in the current study, our data are well in line with previous reports showing that low-intensity exercise with BFR is superior to low-intensity exercise alone (Segal et al., 2015). Especially the effects on pain perception during and after the prehabilitation protocol makes BFR training particularly interesting for patients with degenerative joint diseases. Our results show a significant reduction in pain during the 6-week prehabilitation period in patients with terminal gonarthrosis. These findings are well in line with the literature, showing significant reductions in pain in traumatic and degenerative triggered joint diseases by BFR (van Cant et al., 2020; Pitsillides et al., 2021). Since pain is one of the main symptoms in gonarthrosis (Jones et al., 2000) and can serve as a predictor of mortality during a 10-years post-surgery period (Dennis et al., 2016), the present results of pre- and postoperative pain reduction through BFR-prehabilitation are of particular importance.

Although, scientific knowledge about the underlying mechanisms and safety of BFRE is rising in the last years, a regular implementation of BFRE in clinical settings is not given at present. Therefore, it is important to note that the BFR application in this study was done without evoking adverse effects or leading to a drop out by concurrent rising patient compliance to this training method. The study protocol consists of an individual approach in BFR application (Patterson et al., 2019) by measuring the LOP before prehabilitation and applying a pneumatic-controlled pressure individualized to the LOP of the patient with a tourniquet of 11.5 cm wide. Regarding the necessary BFR pressure to induce muscular effects, there is an ongoing debate. While results from Ilett and colleagues (Ilett et al., 2019) show that most beneficial acute effects are induced by a pressure application of ≥60% of LOP, Counts et al. (2016) reported by regular application, that pressures of 40% LOP are also sufficient for hypertrophy effects. In our study, a BFR pressure of 40% LOP was applied to ensure high patient compliance to the exercise. Based on the positive results of this study, it can be concluded that in case of a reduced training status of the muscles of a subject, low BFR pressures such as 40% LOP are sufficient enough to induce significant effects on muscle mass and strength. Based on this individualized approach of BFRE, it is possible to provide a safe, patient compliant and efficient training for patients with end-stage gonarthrosis.

Although BFRE has demonstrated positive results in postoperative rehabilitation after anterior cruciate ligament reconstruction or conservative therapy of degenerative diseases of the knee joint (Charles et al., 2020), its use as a prehabilitation strategy in degenerative joint diseases has not been previously investigated. As the participating patients of the prehabilitation groups underwent surgery with enhanced muscle mass and strength, we could thereby also address the issue of the “better in, better out” principle.

First of all, the present study reported the classical course of regeneration of skeletal muscle mass and strength after primary TKA in all groups with an initial decrease after surgery (Table 3, 6), and inverse improvement in perceived pain (Figure 3) (Mizner R. L. et al., 2005). However, even if the BFR-group follows this trend as well, our results show that the drop in muscle mass and strength 3 months after surgery does not fall below the baseline values. In comparison to the AC-group, which showed a reduction in muscle mass and strength to baseline, or CON, which partly dropped below the baseline levels, patients of the BFR-group remain consistently better 3 months after TKA than before the start of the prehabilitation-phase (Table 3, 6). Since these results are associated with an improved CRT 3 months post-surgery of the BFR-group in comparison to the other groups, it can be concluded that prehabilitation with BFRE shows a supportive impact on muscle and functional maintenance after TKA surgery. These changes lead to improved outcomes in the early rehabilitation phase which is also illustrated by higher scores in the KOOS (Figure 3).

After 6 months post-surgery, all groups showed a significant increase in muscle strength in comparison to 3 months post-surgery. However, the BFR-group exclusively achieves additional improvements in muscle mass and strength to baseline values already 6-month after surgery (Table 3, 6). Whereas no significant change in muscle mass and strength to baseline for the AC group was revealed, CON showed significant decreased outcomes after 6 months (Table 3 and 6). These findings are well in line with previous literature, illustrating persistent reductions in muscle mass and strength post TKA for patients without prehabilitation (Bade et al., 2010). Our results suggest that prehabilitation with BFRE enables patients to recover postoperative muscular deficits faster than control groups and were able to improve skeletal muscle mass, strength and disbalances to the contralateral leg within the first 6 months postoperatively. This result stands in contrast to previous prehabilitation concepts, which showed only a minor impact on postoperative rehabilitation (Moyer et al., 2017).

## Limitations

The following limitations should be considered when interpreting our findings. Firstly, the methodology of measuring muscle mass by extremity circumference used in this study should be considered as an index of change in muscle size. Since these kinds of measurements includes soft-, adipose- connective- and muscle-tissue, only an estimation of the muscle mass and its change in the course of the study can be done. Future studies should use more valid methods of muscle mass calculation, such as body composition analysis by DXA measurements or MRI scans. Secondly, a possible interference in our results could be caused by a missing matching of the groups to baseline characteristics such as level of physical activity, preoperative muscular deficits, or leg-dominance. Thirdly, there is a possible risk of attention bias, as prehabilitation groups had more visitations to supervisors through the weekly training than the CON, which may have influenced the results preoperatively. Fourthly, level of activity and intensity of activity of the patients after the surgery was not recorded. Future studies should try to monitor postoperative patient activity to get valid data about the effects of prehabilitation on postoperative daily activity.

## Conclusion

The present study is the first one describing the supporting impact of BFRE on skeletal muscle mass, strength, subjective pain perception and QoL pre-as well as post-TKA surgery. BFR prehabilitation appears to be a safe, patient compliant, easy-to-perform and effective tool to improve pre-as well as postoperative clinical outcomes and patient satisfaction in TKA. In a highly standardized clinical intervention such as TKA, BFR prehabilitation allows to prepare the patient physical capacities in the best possible way for surgery. Furthermore, in contrast to previous findings, the present study shows that prehabilitation with BFR is able to support rehabilitation after primary TKA in a “better in, better out”-manner.

## Data Availability

The original contributions presented in the study are included in the article/Supplementary Material, further inquiries can be directed to the corresponding author.
